# Association between Perceived Protection Motivation, Preventive Behaviors, and Biomarkers among Diabetic Patients in Rural Areas of Thailand

**DOI:** 10.3390/clinpract13060126

**Published:** 2023-11-13

**Authors:** Rattanaporn Yongpet, Katekaew Seangpraw, Parichat Ong-Artborirak

**Affiliations:** 1 School of Public Health, University of Phayao, Phayao 56000, Thailand; 64224525@up.ac.th; 2 Department of Research and Medical Innovation, Faculty of Medicine Vajira Hospital, Navamindradhiraj University, Bangkok 10300, Thailand; ong.artborirak@gmail.com

**Keywords:** protection motivation theory, preventive behaviors, diabetes, glycemic control, biomarkers

## Abstract

The prevalence of diabetes mellitus (DM) is increasing rapidly worldwide. Without proper management, DM can have serious complications. We aimed to investigate the association between protection motivation theory (PMT) constructs, self-care behaviors, and biomarkers among diabetic patients in a community in northern Thailand. A cross-sectional study was conducted. Simple random sampling was used to select 422 subjects from patients enrolled in primary care in Chiang Rai Province. Data were collected using questionnaires and blood sampling to measure fasting blood sugar (FBS) and glycated hemoglobin (HbA1c). Pearson’s correlation and multiple linear regression were used to analyze the data. The diabetic subjects’ age was 62.25 years (standard deviation [SD] = 8.90), and the duration of diabetes was 9.07 years (SD = 7.23). Positive correlations were found between the DM knowledge score and the PMT score (*r* = 0.812, *p* < 0.01) and between the PMT score and the preventive behavior score (*r* = 0.817, *p* < 0.01). The preventive behavior score was negatively correlated with FBS (*r* = −0.319, *p* < 0.01) and HbA1c (*r* = −0.625, *p* < 0.01) and significantly associated with income (*B* = 0.15) and the PMT score (*B* = 0.71), accounting for 67.0% of the variance. Age (*B* = −0.73), sleep problems (*B* = 10.71), and the preventive behavior score (*B* = −1.15) were statistically significantly associated with FBS (*R*^2^ = 14.3%). Four variables, the female gender (*B* = −0.26), being married (*B* = −0.24), sleep problems (*B* = 0.42), and the preventive behavior score (*B* = −0.09), were statistically significantly associated with HbA1c levels (*R*^2^ = 41.6%). Health education programs should primarily target awareness of disease severity, complications, and self-efficacy enhancement to generate intention and behavior change. This may delay or reduce the occurrence of diabetes-related complications in people with type 2 diabetes.

## 1. Introduction

Type 2 diabetes mellitus (T2DM) is a commonly found condition and is one of the major health threats to the world population [1]. According to the International Diabetes Federation, in 2017, there were 463 million people with diabetes worldwide, and it is projected that by 2045, the number of people with diabetes will increase to 629 million [1]. In addition, data on the diabetes situation in the Western Pacific in 2017 indicated that Thailand then had 4.4 million people with diabetes, and the mortality rate from diabetes was 21.96 per 100,000 people [1,2]. This was the fourth highest number after China, India, and Japan [2]. In general, the harmful effects of diabetes include microvascular complications, including nephropathy, retinopathy, and peripheral neuropathy, and macrovascular complications, such as coronary artery disease, peripheral arterial disease, and stroke [3]. People with T2DM, particularly those with two or more diabetes complications, have substantially greater societal costs and a lower quality of life [4]. This could be mitigated by early detection and prevention of diabetes complications [5].

Chiang Rai is a northern province in Thailand with a mixture of lifestyles, cultures, and customs among its rural communities. According to a report by the Chiang Rai Provincial Health Data Center, the number of new cases of diabetes increased in 2022–2023, equal to 6758.97, 7169.99, and 7267.50 per 100,000 population [6]. These diabetic patients are prone to complications, especially kidney complications [6]. In 2021, 3.18% of new cases of diabetic patients were diagnosed with chronic kidney disease, and this increased to 6.59% in 2022 [6]. In addition, Chiang Rai province is one of the 10 northern provinces with diabetic patients who die from chronic kidney complications [6]. Previous research has found that risk factors for diabetic patients who are prone to kidney complications include an uncontrolled blood sugar level, the duration of diabetes, a family history of diabetes with renal complications, smoking, alcohol consumption, and obesity [7]. Some studies indicate that chronic kidney disease can occur in diabetic patients who are unable to control their blood sugar levels [7,8]. A biomarker of renal failure in diabetic patients is elevated blood glucose levels (HbA1c), which cause endothelial damage, resulting in decreased glomerular filtration [8].

Protection motivation theory (PMT) has been applied to a wide range of behavioral health practices, and it has been widely used as a framework to predict protective behaviors and assess determinants of and willingness to follow prevention and treatment practices [9]. The theory proposes that individuals exhibit healthy behaviors or are willing to perform these behaviors based on two threat appraisals (the perceived severity and susceptibility of the condition) and three coping assessments (the perceived efficacy of the behavior, perceived barriers to the behavior, and perceived self-efficacy to perform the action) [10,11]. A previous study explained that motivation is the best predictor of health behaviors related to self-care, such as physical activity and diet, in patients with non-chronic diseases [12]. Further studies have explained that PMT constructs correlate with the intention to exercise and significantly predict exercise intentions in patients with T2DM [13]. A preliminary pilot study found that the use of PMT in diabetic awareness assessment is sparse and limited in rural areas. Therefore, the purpose of this study was to assess perceived health, preventive behaviors, and biomarker indicators among diabetic patients in a community in northern Thailand. The results of this study will be used to plan for health promotion and to create a program to control blood sugar levels that is suitable for a rural context to prevent the severity and complications that cause death or disability among diabetic patients.

## 2. Materials and Methods

### 2.1. Study Design

This cross-sectional study was conducted in Khun Tan District, Chiang Rai Province, in northern Thailand. The duration of the study was 4 months, from October 2022 to January 2023. Purposive sampling was employed to select the study area in Khun Tan District, where the head of the public health administration gave permission and was willing to cooperate in conducting the research. Based on the literature review, few studies have used theoretical concepts regarding PMT among diabetic patients in rural areas of northern Thailand; therefore, this research applied the PMT concept as a conceptual framework.

### 2.2. Participants

Simple random sampling was employed to select two sub-districts: Pa Tan and Ta. A sample size calculation based on Cochran’s formula was applied, where n represented the sample size, Z represented the confidence level set by the researcher (at the 95% confidence level = 1.96), and E represented the maximum error that occurred, which was 0.05. Based on this calculation, we obtained a sample size of 384 people to participate in the study. However, to prevent discrepancies in data collection and to reduce the problem of incomplete or fewer than required questionnaire responses (missing data), we increased the sample size by 10%; therefore, the total number of participants was 422 persons. The participants were selected using simple random sampling from a list of patients enrolled in primary health care centers during the years 2021–2022 and recorded in the Hosxp system program of Khun Tan Hospital and the Chiang Rai Provincial Health Data Center. The inclusion criteria comprised male and female patients with T2DM who (a) were aged 35 years and over without kidney complications, (b) had a glomerular filtration rate (GFR) > 60 mL/min over the course of one year, (c) had cumulative blood glucose (HbA1c) ≥ 7% and cumulative blood glucose (FBS) ≥ 126 mg/dL over a one-year period, (d) had lived in the area for at least 1 year, (e) were able to communicate in the local language, and (f) voluntarily participated and signed a written consent form prior to joining the research. People with cognitive or mental disorders, dementia, or blindness were excluded from the study.

### 2.3. Data Collection

Before conducting the research, an announcement was made to recruit five research assistants in the sub-district area, including two nurses, two public health academics, and one village health volunteer. The research assistants were required to be able to communicate in the local language and access the participants as quickly as needed. Before proceeding, a meeting between the researcher and assistants was organized with the aim of clarifying the purpose of the study, the data collection process, techniques for completing the questionnaires, the participants’ rights and privacy, re-checking the data, and related research matters step by step to ensure that everyone understood the process and was moving in the same direction. The researcher translated the official language into the local language for the research assistants to ensure that the meanings of the questionnaires stayed the same. For the data collection process, the researcher coordinated with the persons in charge of the research areas by liaising with Khun Tan Hospital and the health-promoting hospital in the sub-district. After obtaining written consent to conduct the survey, the questionnaires were distributed to the participants. The questionnaires were self-administered by those who could read and write; for persons who could not read or write, the research assistant conducted a face-to-face interview to help check off the questions. The researcher and his assistant assigned interviews for the participants at the Health Promoting Hospital in the sub-district between 09.00 and 17.00 or when convenient. The duration of the interviews was approximately 20–30 min per person.

### 2.4. Research Instruments

The quantitative questionnaire was developed from the literature review and adapted for research in a rural area of northern Thailand [10,14]. The questionnaire was validated by three external experts in the relevant fields of behavioral health, internal medicine, and public health. Part 1 of the questionnaire contained questions about the participants’ general characteristics (i.e., gender, marital status, education level, occupation, chronic diseases, cigarette smoking, alcohol consumption, and sleeping quality) and fill-in questions, including age, body mass index (BMI), income in THB, and duration of diabetes. Part 2 of the questionnaire covered knowledge [14] about diabetes and kidney complications, in which the nature of the questions concerned the mechanisms, pathogenesis, causes, symptoms, and complications of the disease with 15 items. The question type was multiple-choice with “yes” and “no” answers. The scoring criteria were set as 1 point for a correct answer and 0 points for an incorrect answer. The total score ranged from 0 to 15 points and was divided into three levels: low (0–8 points), moderate (9–11 points), and high (≥12 points). Parts 3, 4, 5, and 6 of the questionnaires concerned PMT structures [10]. These included noxiousness (10 items) (for example: diabetes patients with blood sugar levels that are higher than normal for several months in a row will have kidney complications; diabetes affects kidney complications and leads to kidney failure), perceived probability (10 items), self-efficacy (10 items), and response efficacy of behavioral responses for renal complication prevention (10 items). The question responses had three levels, using a Likert scale of “agree,” “not sure,” and “disagree” for both positive and negative questions, and the scores were divided into three levels: high scores ≥80% (≥24 points), moderate scores between 60% and 79% (18–23 points), and low scores of less than 60% (≤17 points). The final part of the questionnaire focused on preventive behaviors [14] toward diabetic nephropathy complications with questions on food consumption (10 items), exercise (5 items), medication intake (5 items), and stress management (5 items), totaling 25 items. The questions contained 11 negative and 14 positive questions, using a scale with three rating levels: “never practiced,” “rarely practiced (1–3 times/week),” and “regularly practiced (4–7 times/week).” The scores were divided into three levels: high scores ≥80% (≥60 points), moderate scores between 60% and 79% (45–59 points), and low scores, which were less than 60% (≤44 points). A tryout of the questionnaires was conducted among 30 participants who had characteristics similar to those in the study. The reliability of part 2 of the questionnaire using the Kuder–Richardson Formula was 0.820. The reliability of parts 3–7 of the questionnaire using Cronbach’s alpha coefficient was 0.71, 0.73, 0.71, 0.74, and 0.70, respectively.

For the biomarker indicators, after the participants voluntarily agreed to participate in the study, they were given clarification and detailed information about the objective and preparation for drawing a blood sample. They had to prepare the day before the blood was drawn and abstain from food and drink for at least 12 h after 8:00 p.m. They were scheduled to meet at the local health-promoting hospital at 7:00 am. An amount of 5 cc of blood for the FBS and HbA1C tests was drawn from the patients by a medical technician or a professional nurse. The blood samples were sent to the Khun Tan Hospital laboratory, and the results were reported through the Hosxp system of Khun Tan Hospital.

### 2.5. Statistical Analysis

Statistical analysis was carried out using a computer software program. Descriptive statistics, such as frequency, mean, percentage, standard deviation (SD), minimum, and maximum, were used to describe the population’s socio-demographic data. The Pearson correlation coefficient (r) was used to investigate the relationships between variables such as knowledge, PMT constructs, preventive behaviors, and biomarkers (FBS and HbA1C). Multiple linear regression with the backward elimination technique was used to identify factors such as general characteristics and preventive behaviors associated with the biomarkers (FBS and HbA1C). The level of statistical significance was set at 0.05.

## 3. Results

Of the 422 participants, more than half were female (63.3%), and 41.2% were aged over 65 years (mean ± SD = 62.25 ± 8.90, min–max = 35–85). Regarding general demographic information, most participants were married (69.2%), had completed primary education or less (74.9%), were currently working (76.5%), and had insufficient income (66.8%). As for health status, in the past month, participants had smoked (5.9%), consumed alcohol (29.1%), and experienced stress and anxiety (8.1%) and sleep problems (19.9%). Most participants (73.7%) had other chronic diseases, including hypertension (57.8%), blood lipids (33.1%), and osteoarthritis (10.9%). Most had diabetes with a duration of <5 years (39.6%, (mean ± SD = 9.07 ± 7.23, min–max = 1–35). Nearly half were obese, with a BMI of 23.0–24.9 kg/m^2^, (44.0%, mean ± SD = 24.63 ± 3.89, min–max = 14.52–41.52). In terms of FBS, more than half had levels ≥126 mg/dL (61.4%, mean ± SD = 140.36 ± 36.83, min–max = 73–405), and almost all had HbA1c levels between 7% and 8.9% (81.5%), as shown in Table 1.

Table 2 presents the mean knowledge scores on diabetes and kidney complications; almost half of the participants obtained a score at a moderate level (43.6%, mean = 10.09, SD = 2.62, min–max = 3–15). Regarding PMT constructs, when classifying the components, we found that noxiousness was low (75.1%) (SD = 2.63), perceived probability was moderate (57.3%) (SD = 2.45), self-efficacy was high (38.4%) (SD = 4.63), and response efficacy was high (45.3%) (SD = 3.65). In terms of preventive behaviors, the mean score was 47.41 (SD = 9.99) points, and most scores were moderate (61.4%) or low (33.6%).

Table 3 presents the results of the correlation analysis among the variables of each PMT construct. All the variables had statistically significant positive correlations (*p* < 0.01). The highest correlation was found between response efficacy and self-efficacy (*r* = 0.724), whereas the correlation between noxiousness and response efficacy was the lowest (*r* = 0.478).

Table 4 presents the results of relationship analysis between knowledge, PMT constructs, preventive behaviors, FBS, and HbA1c. Positive correlations were found between the knowledge score and the total PMT score (*r* = 0.812, *p* < 0.01), as well as between the total PMT score and the preventive behavior score (*r* = 0.817, *p* < 0.01). The preventive behavior score was negatively correlated with FBS (*r* = −0.319, *p* < 0.01) and with HbA1c (*r* = −0.625, *p* < 0.01).

Table 5 presents the results of multiple linear regression analysis using the backward elimination method. Two variables were found to have statistically significant relationships with preventive behaviors (*p* < 0.05): income (*B* = 0.15) and total PMT score (*B* = 0.71). These variables could explain why variations in preventive behaviors accounted for 67.0%. The factors correlated with FBS (*R*^2^ = 0.143, *p* < 0.05) were age (*B* = −0.73), sleep problems (*B* = 10.71), and the preventive behavior score (*B* = −1.15). Moreover, variables including the female gender (*B* = −0.26), being married (*B* = −0.24), sleep problems (*B* = 0.42), and the preventive behavior score (*B* = −0.09) were significantly correlated with HbA1c (*R*^2^ = 0.416, *p* < 0.05).

## 4. Discussion

The findings of our study suggest that cognitive variables, PMT constructs, and preventive behaviors are statistically significantly correlated with biomarkers (FBS and HbA1c) in diabetic patients. The participants in our study had moderate levels of knowledge about diabetes and nephrotic complications. The questions on which the participants received low scores regarding diabetes knowledge included that the disease is caused by impaired kidney function, poor blood glucose control, causes susceptibility to infection, and that swelling of the arms, legs, face, and torso is a sign that diabetic patients are beginning to have kidney dysfunction. The results showed that knowledge was significantly correlated with preventive behaviors, FBS, and HbA1c. A previous study mentioned that having knowledge of diabetes can help with early detection and reduce complications; furthermore, the level of knowledge among at-risk populations is associated with better health outcomes [15]. A statistically significant relationship between DM knowledge and self-care behaviors was found among T2DM patients in Northern Thailand (*r* = 0.344) [16]. According to a study of diabetic Koreans, cognitive factors were important, and individuals with a high knowledge of kidney disease were more likely to have good preventive and self-care behaviors to prevent complications [17]. Similarly, a literature review found that increasing a person’s knowledge has the effect of changing their attitudes and leads to self-beneficial actions, so that promoting diabetes knowledge and self-practice among patients should be encouraged to increase their awareness, resulting in effective prevention and control of diabetes risk factors [18]. These results emphasize the importance of diabetes knowledge in educating patients with T2DM, especially on diabetes complications, both traditional and emerging complications, and effective treatments, including lifestyle changes, medications, and novel treatments such as bariatric surgery [19].

### 4.1. Correlations between PMT Constructs

An important finding of our study was the analysis of PMT constructs by their components. The results were low noxiousness (75.1%), moderate perceived probability (57.3%), high self-efficacy (38.4%), and high response efficacy (45.3%). When analyzing the relationships between the variables in each PMT construct, all the variables were found to have statistically significant positive correlations (*p* < 0.01). We also found the highest correlation between response efficacy and self-efficacy (*r* = 0.724). The results of this study are consistent with a previous systematic review, which concluded that PMT is moderately successful in predicting attention and behavior related to health and safety in a variety of contexts, such as cigarette smoking, alcohol consumption, nutrition, and exercise [20,21]. PMT is a useful theory for predicting health and preventive behaviors; moreover, meta-analyses have reported significant effects for all PMT variables [9,20]. Our results are comparable to those of previous studies included in a meta-analysis of health behaviors among the general population. The findings showed that coping appraisal variables, especially self-efficacy, gave the strongest predictions of protective motivation (intention) and preventive behavior [20]. Consistent with previous studies, PMT constructs were found to be able to predict 60% of the variance in intentions for physical activity, and an increase in physical activity intention and self-efficacy scores could increase the likelihood of higher levels of physical activity by 3.4 and 1.5 times (OR = 3.39, 1.54), respectively 21]. Similar to our study, in a study of diabetic foot care behaviors in Egypt, PMT constructs strongly predicted self-efficacy behavior (*p* = 0.015), perceived probability (*p* = 0.013), and intention to practice foot care (*p* = 0.021) [11].

### 4.2. Association between General Demographic Information, PMT Constructs, and Preventive Behaviors

For preventive behaviors, our study showed that more than half of the participants obtained moderate and low scores (61.4% and 33.6%, respectively). Moreover, when analyzing the correlation between PMT constructs and preventive behaviors, it was found to be statistically significant (*p* < 0.01). Considering each question for which the participants had low scores, they included the following: consuming food and drink that contain high fat, sodium, and sugar, including rice, pork leg, pork rind, fried food, pork skin, sugary water, and fruit juices; less than 20 min of exercise, e.g., walking, jogging, and swinging arms; adjusting insulin doses by reducing, increasing, or stopping the drug on their own; and having slept less than 6 h. In addition, the participants in our study had lived with diabetes for less than 5 years (39.6%), and most were early-old adults. However, most of them may not have had much experience of taking care of their own health in terms of the disease and its complications; diabetes is a chronic disease with renal complications that requires a long process of treatment and knowledge for one to perform self-care and health-protective behaviors [22]. This is similar to the health concept in Pender’s theory, in which biological factors such as age, health status, socio-cultural norms, and certain characteristics and lifestyles of individuals are risk factors for their health status that determine change in patients’ self-care behaviors [23]. Furthermore, the PMT concept suggests that individuals exhibit healthy behaviors and/or have the intention to perform these behaviors based on their cognitive assessment processes [10]. Studies using PMT have been conducted on diabetic patients to predict their physical activity [13,21], treatment compliance [24], and diabetes prevention practices [11]. Similar to previous studies, in our study, we found that more than half of the participants obtained low and moderate scores for self-care behaviors (62.5%) [25]. In many other studies, most patients obtained low scores for self-care behavior [26,27,28,29].

When analyzing the relationships using multiple regression, we found that income and PMT constructs had statistically significant positive correlations with preventive behaviors (*R*^2^ = 67.0%). Similarly, a previous study conducted among T2DM patients in urban western China found a significant difference in self-management behavior scores according to income [30]. Another study conducted among ethnic minorities in southwest China found that those living in communities with higher income levels had an increased likelihood of compliance with prescribed medicines [31]. Concerning variables, PMT constructs were associated with preventive behaviors, corresponding with previous research suggesting that cognitive behaviors related to diabetic patients’ compliance with PMT constructs influenced health attention and helped change some behaviors effectively [13]. Our results also correspond with previous studies indicating that there is a relationship between threat appraisal (noxiousness and perceived probability) and intention to engage in physical activity, and that coping responses, including response efficacy and self-efficacy expectancy, are strong predictors of motivation [13,32]. Our results are also consistent with several studies showing that perception and intrinsic motivation are significantly positively correlated with diabetic patients’ self-care behaviors [12,33].

### 4.3. Association between General Demographic Information, Preventive Behaviors, FBS, and HbA1C

The analysis of key variables in this study, namely age, sleep problems, and preventive behaviors, found statistically significant associations with FBS levels (*R*^2^ = 14.3%), meaning that diabetic patients were more likely to lose control of their blood sugar levels with increasing age. However, this may be because older adults are experiencing life changes that are critical and that make it difficult to adapt to the environment [27]. Therefore, diabetic patients feel more dependent on others and less likely to practice self-care management of the disease, causing them to experience a decline in health and to have difficulty controlling blood sugar levels back to normal [27]. This is consistent with our literature review, which found that it becomes difficult to control blood glucose with increasing age because the insulin secretion of pancreatic beta cells is reduced and insulin resistance increases [34,35]. Studies have shown that in diabetic patients, sleeping disorders such as short sleep duration (<6 h), snoring, and sleeping difficulty are predictive factors for T2DM and hinder patients’ lives [36,37]. Breathing disorders have been associated with sleep problems, FBS, and insulin resistance [36,37]. This is also similar to a recent study that found FBS levels to be inversely correlated with self-care behaviors among older Thai adults with T2DM (*r* = − 0.34) [29]. Poor glycemic control has been shown to be associated with later complications [27,38].

The final variables to be discussed in our study are biomarker indicators. By using multivariable analysis, we found that the female sex, marital status, sleep problems, and preventive behaviors were significantly associated with HbA1c level (*R*^2^ = 41.6%). Our results showed that females were more likely to lose control of their blood sugar than males. Our findings are consistent with a previous study that found a significant difference in HbA1c levels between male and female diabetic patients in the United States (8.1% vs. 7.6%) [39]. Sex also had a significant influence on HbA1c levels in people with T2DM in rural China [40]. HbA1c levels in Chinese adults with no prior diagnosis of DM were significantly higher in the male group than in the female group [41]. Among diabetes patients in northwest Ethiopia, married subjects had a 55% lower likelihood of having sub-optimal glycemic levels compared to single subjects [42]. Being married was found to be associated with lower HbA1c values among adults without pre-existing diabetes [43].

In addition, previous reviews have suggested that sleep disturbances have been reported in more than one-third of people with T2DM, which may be attributable to fear of poor blood glucose control and diabetic complications [44]. Some studies have reported an inverse association between subjective sleep disturbances and poor glycemic control in people with T2DM [45]. Sleep duration variability has been strongly associated with HbA1c (*B* = 0.239; *p* = 0.002; *R*^2^ = 4.9%), and the duration of sleep has also been correlated with HbA1c, with a 6.0% overlap between the two [46]. One study also indicated that insomnia (39%) was more common in people with T2DM compared to the general population, and that sleep quality was affected in patients with prolonged hyperglycemia [47,48]. Consistently with several studies, we found that self-care behaviors were statistically significantly associated with HbA1c (*p* < 0.05) [25,49]. Self-care practices were the strongest indicator of HbA1c among patients with type 2 diabetes in Iran (β = −0.59) [30]. According to a recent study [31], HbA1c levels are inversely related to self-care behavior scores in older Thai adults with T2DM (*r* = − 0.40). Therefore, self-care behavior is essential for patients with T2DM because it helps patients to control their blood sugar levels and have better health-promoting practices.

### 4.4. Limitations

Our study’s limitations include, first, low sample size. This was because the inclusion criteria focused on participants with HbA1c ≥ 7% and cumulative FBS of ≥126 mg/dL during a period of one year. In future studies, the sample size should be greater, data should be collected for a period of one month after the examination of biomarker indicators, and several key variables should be included for further analysis. Second, this cross-sectional study precludes causal conclusions and can only link PMT constructs, preventive behaviors, FBS, and HbA1c in the selected study area. Third, the sample was a cohort of patients in Khun Tan District, Chiang Rai Province, and it is possible that our findings may not be representative of other populations throughout the country. This study highlights the need for caution in extending study results to populations, particularly in rural areas. Finally, the study environment and the cultural, social, and organizational factors of this study may have affected the outcomes. Future studies should focus on exploring PMT constructs for each domain to determine the individual causal factors affecting self-care behavior and clinical indicators about patient complications. In addition, the results of this study should be used to study the effectiveness of health promotion programs to prevent kidney complications in diabetic patients in the future.

## 5. Conclusions

In general, our findings show that many variables are associated with self-care behaviors and influence FBS and HbA1C levels in diabetic patients. Furthermore, the application of PMT constructs can predict a person’s intention to practice healthcare behaviors. Our findings also suggest that response efficacy and self-efficacy can influence individuals’ motivation to prevent disease. As a result, diabetes prevention and health promotion programs should focus on raising awareness of the disease’s severity and increasing self-efficacy in terms of increased self-care expectancy. However, it is important to emphasize that among females with TD2M, supplementary coping strategies, diabetes self-efficacy, sleep, and self-care behaviors may be important to reduce FBS and HbA1c levels and prevent early diabetes complications.

## Figures and Tables

**Table 1:** General characteristics of participants (*n* = 422).

Variables		*n* (%)
Sex		
	Male	155 (36.70%)
	Female	267 (63.30%)
Age		
	35–44 years	11 (2.61%)
	45–54 years	67 (15.88%)
	55–64 years	170 (40.28%)
	≥65 years	174 (41.23%)
	Mean ± SD	62.25 ± 8.90
	Min.–Max.	35–85
Marital status		
	Single/widowed/divorced/separated	130 (30.80%)
	Married	292 (69.20%)
Education		
	Primary school or lower	316 (74.90%)
	Secondary school or higher	106 (25.1%)
Occupation		
	No	99 (23.50%)
	Yes	323 (76.50%)
Financial status		
	Insufficient	282 (66.82%)
	Sufficient	140 (33.18%)
Income in THB		
	Mean ± SD	2726.18 ± 3626.73
	Min.–Max.	500–30,000
Smoking		
	No	397 (94.10%)
	Yes	25 (5.90%)
Alcohol drinking		
	No	299 (70.90%)
	Yes	123 (29.10%)
Stress or anxiety in the past month		
	No	388 (91.90%)
	Yes	24 (8.10%)
Sleep problems in the past month		
	No	338 (80.10%)
	Yes	84 (19.90%)
Chronic diseases in patients		
	No	111 (26.30%)
	Yes	311 (73.70%)
Type of disease (*n* = 311)		
	Hypertension	244 (57.81%)
	Dyslipidemia	140 (33.17%)
	Gout	25 (5.92%)
	Osteoarthritis	46 (10.90%)
	Other (heart disease, COPD, asthma)	12 (2.84%)
Duration of diabetes mellitus		
	<5 year	167 (39.60%)
	5–10 year	122 (28.90%)
	>10 year	133 (31.50%)
	Mean ± SD	9.07 ± 7.23
	Min.–Max.	1–35
Body Mass Index (BMI)		
	Underweight (<18.5 kg/m^2^)	15 (3.55%)
	Normal (18.5–22.9 kg/m^2^)	132 (31.28%)
	Overweight (23.0–24.9 kg/m^2^)	89 (21.10%)
	Obesity (≥25.0 kg/m^2^)	186 (44.07%)
	Mean ± SD	24.63 ± 3.89
	Min.–Max.	14.52–41.32
Fasting blood sugar (FBS)		
	<126 mg/dL	163 (38.60%)
	≥126 mg/dL	259 (61.40%)
	Mean ± SD	140.36 ± 36.83
	Min.–Max.	73–405
Glycated hemoglobin (HbA1C)		
	7.0–8.9%	344 (81.50%)
	≥9.0%	78 (18.50%)
	Mean ± SD	8.15 ± 1.38
	Min.–Max.	7.0–17.2

**Table 2:** The level of knowledge, PMT constructs, and preventive behaviors among participants (*n* = 422).

Variables		*n* (%)
Knowledge		
	Low level (scores 0–8)	118 (27.96%)
	Moderate level (scores 9–11)	184 (43.60%)
	High level (scores 12–15)	120 (28.44%)
	Mean ± SD	10.09 ± 2.62
	Min.–Max.	3–15
Noxiousness		
	Low level (scores 0–17)	317 (75.1%)
	Moderate level (scores 18–23)	105 (24.9%)
	High level (scores 24–30)	0 (0.0%)
	Mean ± SD	15.77 ± 2.63
	Min.–Max.	12–23
Perceived Probability		
	Low level (scores 0–17)	180 (42.70%)
	Moderate level (scores 18–23)	242 (57.30%)
	High level (scores 24–30)	0 (0.0%)
	Mean ± SD	17.68 ± 2.45
	Min.–Max.	13–22
Self-Efficacy		
	Low level (scores 0–17)	101 (23.9%)
	Moderate level (scores 18–23)	159 (37.7%)
	High level (scores 24–30)	162 (38.40%)
	Mean ± SD	21.86 ± 4.63
	Min.–Max.	14–29
Response Efficacy		
	Low level (scores 0–17)	91 (21.60%)
	Moderate level (scores 18–23)	140 (33.20%)
	High level (scores 24–30)	191 (45.30%)
	Mean ± SD	21.75 ± 3.65
	Min.–Max.	13–28
Preventive behaviors		
	Low level (scores 0–44)	142 (33.60%)
	Moderate level (scores 45–59)	259 (61.40%)
	High level (scores 60–75)	21 (5.00%)
	Mean ± SD	47.41 ± 9.99
	Min.–Max.	25–61

**Table 3:** Correlation coefficients (*r*) between protection motivation theory constructs among participants (*n* = 422).

PMT Construct	1	2	3	4	5
1. Total PMT	1				
2. Noxiousness	0.755 **	1			
3. Perceived Probability	0.856 **	0.654 **	1		
4. Self-Efficacy	0.919 **	0.577 **	0.713 **	1	
5. Response Efficacy	0.858 **	0.478 **	0.643 **	0.724 **	1

** Correlation is significant at the 0.01 level (2-tailed).

**Table 4:** Correlation coefficients (*r*) between knowledge, protection motivation theory constructs, preventive behaviors, and biomarkers among participants (*n* = 422).

Variables	1	2	3	4	5
1. Knowledge	1				
2. PMT constructs	0.812 **	1			
3. Preventive behaviors	0.787 **	0.817 **	1		
4. FBS	−0.295 **	−0.397 **	−0.319 **	1	
5. HbA1C	−0.586 **	−0.696 **	−0.625 **	0.478 **	1

** Correlation is significant at the 0.01 level (2-tailed).

**Table 5:** Factors associated with study outcomes among participants (*n* = 422) using multiple linear regression with backward elimination technique.

Outcome	Factor	*B*	S.E.	Beta	*p*-Value	95% CI	*R* ^2^
Preventive behaviors	Income in THB 1000	0.153	0.077	0.056	0.048	0.001, 0.305	67.0%
	Total PMT score	0.712	0.024	0.818	<0.001	0.664, 0.760	
FBS	Age in years	−0.734	0.189	−0.177	<0.001	−1.105, −0.363	14.3%
	Sleep problems	10.708	4.198	0.116	0.011	2.456, 18.961	
	Preventive behavior score	−1.146	0.168	−0.311	<0.001	−1.475, −0.817	
HbA1C	Sex	−0.258	0.11	−0.09	0.019	−0.474, −0.043	41.6%
	Marital status	−0.239	0.114	−0.08	0.037	−0.463, −0.015	
	Sleep problems	0.424	0.131	0.122	0.001	0.167, 0.680	
	Preventive behavior score	−0.087	0.005	−0.631	<0.001	−0.098, −0.077	

Notes: variables: sex (0 = male, 1 = female); marital status (single/widowed/divorced/separated = 0, married = 1); sleep problems (no = 0, yes = 1); income, age, PMT constructs, preventive behavior, fasting blood sugar (FBS), glycated hemoglobin (HbA1c) = continuous data.

## Data Availability

The data presented in this study is available on request from the corresponding author.
